# Consensus Statement for the Prescription of Pain Medication at Discharge after Elective Adult Surgery

**DOI:** 10.1080/24740527.2020.1724775

**Published:** 2020-03-08

**Authors:** Hance A. Clarke, Varuna Manoo, Emily A. Pearsall, Akash Goel, Adina Feinberg, Aliza Weinrib, Jenny C. Chiu, Bansi Shah, Salima S. J. Ladak, Sarah Ward, Sanjho Srikandarajah, Savtaj S. Brar, Robin S. McLeod

**Affiliations:** a Department of Anaesthesia and Pain Management, Toronto General Hospital, Toronto, Ontario, Canada; b Transitional Pain Service, Department of Anaesthesia and Pain Management, Toronto General Hospital, Toronto, Ontario, Canada; c Department of Anaesthesia, University of Toronto, Toronto, Ontario, Canada; d University of Toronto Centre for the Study of Pain, University of Toronto, Toronto, Ontario, Canada; e Department of Surgery, University of Toronto, Toronto, Ontario, Canada; f Department of Pharmacy, North York General Hospital, Toronto, Ontario, Canada; g Faculty of Nursing, University of Toronto, Toronto, Ontario, Canada; h Department of Surgery, St. Michaels Hospital, Toronto, Ontario, Canada; i Department of Anaesthesia, North York General Hospital, Toronto, Ontario, Canada; j Department of Surgery, Mount Sinai Hospital, Toronto, Ontario, Canada

**Keywords:** postoperative pain management, postsurgical opioid prescribing, clinical practice guideline, best practices in surgery, harm reduction

## Abstract

This Consensus Statement provides recommendations on the prescription of pain medication at discharge from hospital for opioid-naïve adult patients who undergo elective surgery. It encourages health care providers (surgeons, anesthesiologists, nurses/nurse practitioners, pain teams, pharmacists, allied health professionals, and trainees) to (1) use nonopioid therapies and reduce the prescription of opioids so that fewer opioid pills are available for diversion and (2) educate patients and their families/caregivers about pain management options after surgery to optimize quality of care for postoperative pain.

These recommendations apply to opioid-naïve adult patients who undergo elective surgery. This consensus statement is intended for use by health care providers involved in the management and care of surgical patients.

A modified Delphi process was used to reach consensus on the recommendations. First, the authors conducted a scoping review of the literature to determine current best practices and existing guidelines. From the available literature and expertise of the authors, a draft list of recommendations was created. Second, the authors asked key stakeholders to review and provide feedback on several drafts of the document and attend an in-person consensus meeting. The modified Delphi stakeholder group included surgeons, anesthesiologists, residents, fellows, nurses, pharmacists, and patients. After multiple iterations, the document was deemed complete. The recommendations are not graded because they are mostly based on consensus rather than evidence.

## RÉSUMÉ

Cette déclaration de consensus fait des recommandations pour la prescription d'analgésiques à la sortie de l'hôpital pour les patients adultes n'ayant jamais pris d'opiacés et qui subissent une intervention chirurgicale non urgente. Elle encourage les prestataires de soins de santé (chirurgiens, anesthésiologistes, infirmières et infirmiers, infirmières et infirmiers praticiens, équipes antidouleur, pharmaciens, professionnels de la santé et stagiaires) à (1) utiliser des traitements non opiacés et à réduire la prescription d'opiacés afin de réduire le nombre de pilules opiacées pouvant être détournées; et (2) à éduquer les patients, ainsi que leurs familles et soignants, sur les options de prise en charge de la douleur après l'opération afin d'optimiser la qualité des soins pour la douleur postopératoire.

Ces recommandations s'appliquent aux patients adultes n'ayant jamais pris d'opioïdes et qui subissent une intervention chirurgicale non urgente. Cette déclaration de consensus est destinée à être utilisée par les prestataires de soins de santé impliqués dans la prise en charge des patients opérés et les soins qui leur sont apportés.

Un processus Delphi modifié a été utilisé pour parvenir à un consensus sur les recommandations. Tout d'abord, les auteurs ont procédé à une de la portée de la littérature afin de déterminer les pratiques exemplaires actuelles et les lignes directrices existantes. À partir de la littérature disponible et de l'expertise des auteurs, une liste provisoire de recommandations a été créée. Ensuite, les auteurs ont demandé aux principales parties prenantes d'examiner et de commenter plusieurs versions préliminaires du document et d'assister à une réunion de consensus en personne. Le groupe des parties prenantes du processus Delphi modifié comprenait des chirurgiens, des anesthésiologistes, des résidents, des fellows, des infirmières et infirmiers, des pharmaciens et des patients. Après de multiples itérations, le document a été jugé complet. Les recommandations n'ont pas été notées car elles étaient fondées sur un consensus plutôt que sur des données probantes.

## Background

### Aim

The aim of this consensus statement is to make recommendations for the prescription of pain medication at discharge from hospital for opioid-naïve adult patients who have undergone an elective surgical procedure. This consensus statement aims to encourage health care providers to

1. Minimize the prescription of opioids and encourage the use of nonopioid therapies for postoperative pain to ensure that fewer opioid-containing tablets are available for diversion.
2. Educate patients and their families/caregivers on pain management options in relation to functional recovery.

### Outcomes of Interest

- Minimize the use of opioids for postoperative pain.
- Maintain an acceptable level of postoperative pain control while minimizing the use of opioids.
- Reduce the number of opioid-containing tablets available for diversion or hoarding.

### Target Population

These recommendations apply to opioid-naïve adult patients who undergo elective surgery.

#### Cautions

Patients who are at high risk of persistent postoperative opioid use (see recommendation 2) should be seen preoperatively by an anesthesiologist or a member of the pain team to develop a perioperative pain management plan. The consensus statement may be utilized in high-risk patients who are opioid naïve, provided that follow-up is arranged postdischarge (ideally by specialized/transitional pain team) given their increased risk of persistent opioid use.

### Intended Users

This consensus statement is intended for use by health care providers involved in the management and care of surgical patients, including surgeons, anesthesiologists, nurses, pain teams, pharmacists, allied health professionals, and residents and fellows.

#### Rationale

The rationale for developing a consensus statement on the management of postoperative pain was largely based on the rising public health concern over the opioid crisis. This can be contrasted with past efforts to avoid opioid use by patients to prevent undesirable side effects. Opioids remain an excellent option for the treatment of acute pain, but if not prescribed responsibly and without proper patient education regarding storage and safe disposal, they can become an easy target for diversion.

To date, the estimated the amount of opioids that remain unused after elective adult surgery can be greater than 80%.^1^ This consensus statement directly impacts the overprescription of opioids following surgery and creates a framework for opioid-naïve patients presenting for elective surgery. It tackles the issue of diversion and provides a proactive solution for the perioperative world.

Though the risk of persistent opioid use and/or the development of an opioid use disorder in elective patients following surgery is low, perioperative practitioners/surgeons must respond to the crisis and act proactively and responsibly by modifying our practices and creating best practices. The authors have taken extreme care in balancing patient well-being with the need for appropriate pain control in the opioid-naïve elective surgery patient (80%–85% of our surgical volumes). As the gatekeepers of opioid access through prescribing, it is imperative that all health care workers involved in the chain of prescribing and dispensing become familiar with safe practices that will protect the public from potential harm or an unnecessarily excessive exposure to opioids.

## Overview of Process

A scoping review of the published literature was conducted to determine whether guidelines for the prescription of opioids after surgery existed. Key words included “opioid,” “multimodal pain management,” “postoperative pain management,” “guidelines,” “clinical practice,” “standards,” and “postoperative.” Though there were many published articles documenting current practice and the need for guidelines; there was limited published literature to guide practice on minimizing the amount of opioids prescribed after elective surgery for opioid-naïve patients. Due to the limited information found, the next step was to review the gray literature, society websites, and hospital websites to determine whether there were guidelines on the prescription of opioids after surgery. This search produced more results.

A master list of all recommendations from each article was compiled by one author (E.P.) and the authors met to discuss the recommendations (or lack thereof). The authors then created draft recommendations based on previously published guidelines and expert opinion.

Once a complete list of draft recommendations was developed by the authors, an email invitation was sent to all staff surgeons, anesthesiologists, and residents at the University of Toronto Departments of Surgery and Anesthesia to be part of the opioid working group. The working group would be responsible for the development of the consensus statement recommendations as well as drafting the supporting evidence. An open call was also sent out in the *Best Practice in Surgery Newsletter*, which goes out to key stakeholders across the province of Ontario. Surgeons and anesthesiologists who responded also recommended pharmacists, nurses, and nurse practitioners from their hospital, who were then invited to join the working group. The working group members are listed as authors on this document.

A modified Delphi process was used to gain consensus on the recommendations due to the lack of available evidence. As part of this process, an invitation was sent via email to all staff surgeons and anesthesiologists associated with the University of Toronto Departments of Anesthesiology and Surgery to invite them to be part of the modified Delphi process. In the invitation, we also requested that surgeons and anesthesiologists extend an invitation to nurses and pharmacists who are interested in postoperative pain management. Lastly, members from the Best Practice in Surgery Patient Advisory Committee were invited to participate. In total, 40 persons agreed to be part of the modified Delphi process. This group included surgeons, anesthesiologists, pharmacists, nurses, and patients.

The modified Delphi process included reviewing several iterations of the consensus document as well as attending an in-person meeting. The first step of the process was for the stakeholders’ group to review a draft consensus statement with recommendations and send feedback prior to the in-person meeting. The working group then reviewed the comments and feedback and made changes as necessary.

The next step was to host an in-person stakeholder meeting that included members of the working group as well as the stakeholders who agreed to be part of the modified Delphi process. The stakeholder consensus meeting was held in November 2018 with participants from University of Toronto–affiliated hospitals as well as other Canadian content experts. The recommendations and supporting evidence were reviewed and discussed in person until consensus was reached or a plan for modification was decided. The document was then further refined based on the discussion and was sent to the same stakeholder group for review. The working group members met again to address the feedback and made any necessary changes.

A near-final draft was then circulated to all end users of the consensus statement, including staff surgeons and anesthesiologists at University of Toronto–affiliated hospitals as well as current residents and fellows from surgery and anesthesiology. Overall, the document was sent to more than 1000 people for review.

Due to the lack of available evidence, these consensus statement recommendations were not graded as is customary for clinical practice guidelines because the majority of the recommendations are based on local consensus and expert opinion.

## Recommendations and Summary of Evidence

1. Patient education
  1.1. Patients and their families and/or caregivers should be provided with written and verbal information on their pain management options preoperatively, including:
    •Expectations regarding functional recovery (returning to meaningful physical activities)
    • Realistic pain management goals (goal is function, not zero pain)
    • Multimodal pain management options (e.g., opioid options, nonopioid options, and nonpharmacological options)
    • Possible interaction of patient’s current medications and their potential interactions with opioids (e.g., sleeping pills, alcohol, benzodiazepines)
    • Risk of potential opioid side effects, overdose, and development of a dependence or addiction
    • Safe opioid use and discontinuation
    • Risk factors for opioid use disorder (history of substance use disorder, depression, anxiety).
    1.1.1. This information should be reinforced and reviewed prior to discharge.

There is limited evidence available to support this recommendation. However, despite the lack of randomized controlled trials supporting the use of patient education to assist with pain management, almost all hospitals provide their patients with patient education materials outlining pain management options. This fact highlights the importance and necessity of informing patients and their families/caregivers preoperatively in order to assist them in managing their expectations postoperatively.

Of the few published studies, most found that preoperative education materials did reduce postoperative pain and reduce patients’ anxiety. Lee et al. conducted a randomized controlled trial with 86 patients undergoing lumbar spinal surgery.^2^ Patients were randomized into the intervention group (received education at intervention) vs. control group (no educational intervention). After controlling for demographics, the authors found that patients who received the educational intervention had significantly lower anxiety scores based on the State-Trait Anxiety Inventory (*P* < 0.001).^2^ The authors also found that patients in the intervention group reported significantly lower pain scores on postoperative day 1 (6.07 vs. 5.28, *P* < 0.001).^2^

There is evidence that psychological interventions can reduce opioid use after surgery, though research in this burgeoning area is in early stages.^3,4^ In addition to receiving presurgical education on pain and opioid use, patients may benefit from presurgical training in psychological skills that can be used to reduce pain intensity, pain unpleasantness, pain catastrophizing, and anxiety. Before surgery and associated pain onset is an ideal time for learning because patients are motivated to reduce postsurgical pain and opioid use and have clearer minds for learning because pain and medications that reduce concentration and increase drowsiness are not yet impacting them. Presurgical psychological interventions can include one or more of the following psychological modalities: mindfulness interventions (including breathing techniques)^5^; clinical hypnosis interventions (including deep relaxation and hypnotic suggestions for lasting comfort)^6^; cognitive behavioral therapy skills training (e.g., single-session behavioral protocols targeted at reducing pain catastrophizing)^7^; acceptance and commitment therapy skills training (i.e., incorporating mindfulness of pain sensations, as well as behavioral activation after surgery based on personally meaningful values and goals)^3^; and dialectical behavior therapy skills (e.g., distress tolerance skills) to reduce implicit or explicit use of opioid medications to regulate emotions in patients who are vulnerable to emotional dysregulation when stressed.^8^ In addition, medical and nursing staff can be trained in medical communication that is mindful of the psychological suggestions implicit in everyday language in order to reduce anxiety and pain intensity after surgery.^9^ The interventions listed above can also be utilized to reduce discomfort and anxiety after surgery while patients are in hospital. Due to the mind–body pathways involved in pain perception, learning and practicing these psychological skills can reduce opioid use while keeping patients more comfortable.

The recommendations that are presented above are the result of reviewing patient education materials and consulting with experts as well as brochures and pamphlets available on hospital websites that outline the information that should be given to patients. The recommendations were also discussed by a group of key stakeholders and consensus was reached. Despite the limited available evidence, there is strong consensus that preoperative education is beneficial for patients and is an essential element of improved pain management with opioid medications after surgery. The Institute for Safe Medication Practices Canada in collaboration with many organizations^10^ and support from the Canadian Patient Safety Institute have developed a patient information sheet on the use of opioids.^11^

The task of educating patients rests with the entire medical team. In some hospitals, residents take the lead; in others, the acute pain service or physician assistants lead. It is at the discretion of each hospital system to decide how information is dispensed and by whom, based on their custom and means.

1.2 Patients should be provided with written and verbal information prior to discharge on the safe storage and disposal of unused opioids in accordance with Health Canada’s recommendations.
  1.2.1. Store opioids in a secure place to prevent theft or accidental exposure.
  1.2.2. Keep opioids out of sight and reach of children and pets.
  1.2.3. Do not keep opioid medications for when they “might” be needed.
  1.2.4. Do not throw opioids into household trash where children and pets may find them.
  1.2.5. Do not flush opioids down the toilet.
  1.2.6. Return expired, unused, or used opioids to a pharmacy for proper disposal.

The Institute for Safe Medication Practices Canada in collaboration with the Canadian Patient Safety Institute developed and released a document in June 2018 to assist providers across Canada in minimizing the potential accidents that may arise when medications, specifically opioids, are improperly stored and/or disposed of.^12^ The most prominent concerns stem from inappropriate use and diversion. Diversion is when drugs from a licit channel (i.e., prescription for pain after surgery) are transferred to an illicit channel (i.e., are stolen, sold, taken) for illegal use or distribution.

It is recommended to store opioids out of sight and reach of children. Studies have shown that children are at increased risk because they may accidentally ingest medications if they have access to them.^13^ It is thus suggested that medications be stored above counter height and ideally in a locked cabinet. Hoarding of opioid medications by patients in anticipation of a day when they “might” be needed should be discouraged, because this will increase the potential for diversion of these tablets.

With regards to safe disposal, it is strongly recommended that unused medication should be returned to the patient’s local pharmacy. Disposing of these medications in other ways has been shown to have adverse effects. If the medications are disposed in the trash, it is still possible for children or pets to access them. In addition, they may be available for diversion. Additionally, flushing medications is a risk to the environment and has health risks.

2. Risk factors for persistent postoperative opioid use
2.1. Preoperatively, patients should be assessed for the following risk factors because they may be at increased risk for persistent postoperative opioid use:
  • Surgical procedures associated with significant nerve damage that may put patients at risk to develop neuropathic pain
  • History of or concurrent anxiety and/or depression and/or high levels of pain catastrophizing and/or posttraumatic stress disorder
  • Use of medications for depression and/or anxiety (e.g., benzodiazepines and selective serotonin reuptake inhibitors)
  • Currently or previously followed and treated for chronic pain under medical supervision
  • History of drug use, smoking, and/or alcohol use disorder (previously or currently)
  • Low socioeconomic status
  • Aged 18–30 years old.

For the hundreds of millions of persons who provide or receive care for major surgical procedures annually, the treatment of acute postoperative pain and its treatment with opioids have become a hot topic.^14^ Opioids remain essential for many patients with respect to treating moderate to severe acute pain after major surgery.^15^ Beyond the common opioid-related side effects (e.g., constipation, pruritus, urinary retention, etc.), some patients go on to develop longer term persistent opioid use,^16^ which negatively impacts their postsurgical quality of life.^17,18^ Long-term use is also associated with increased risks of injury,^19^ cardiac events,^20^ and overdose.^21^

Research aiming to understand the risk factors associated with the conversion from short-term to long-term use of opioids after surgery is ongoing. Studies have identified that the initial opioid prescription is a risk for unwarranted persistent use that increases one’s risk for misuse, opioid-induced hyperalgesia, mood disorders, and endocrine disturbances.^15^ Alam and colleagues reported that patients who received an opioid prescription within 7 days of surgery were 44% more likely to be long-term opioid users at 1 year.^18^ Of note, in the United States, there was a 402% increase in individual opioid use from 1997 to 2007, a significant portion of which stemmed from chronic use after initiation of treatment.^22^ A study of 39,140 patients who underwent major surgery (cardiac, thoracic, abdominal, urologic, and gynecologic procedures) in Ontario from 2003 to 2011 showed that 49.2% received a prescription for opioids at discharge. By 3 months postsurgery, more than 3% of previously opioid-naïve patients continued to fill prescriptions for opioids.^23^ This suggests that for some patients presenting for major surgery, the use of opioids postoperatively for acute pain can carry on well into the postdischarge period.

A significantly higher risk for prolonged opioid use is seen in patients of younger age and lower income, as well as in those with comorbidities such as lung disease, diabetes, and heart failure.^23^ In a recent study, males younger than 30 years of age were identified as a high-risk population for the escalation of their opioid dose postdischarge.^24^ The use of medications such as benzodiazepines, selective serotonin reuptake inhibitors, and angiotensin-converting enzyme inhibitors preoperatively was also associated with increased risk for persistent postoperative opioid use.^23^ Low socioeconomic status has been estimated based on patients’ neighborhood median income in a national census.^25^ It may be helpful for the preoperative assessment or pharmacy team to conduct a best possible medication history to identify patients at risk for potential drug interactions.

A common finding in the literature is that current and previous tobacco use is associated with the risk of intense acute pain and persistent opioid use.^26,27^ Smoking may be an important modifiable risk factor for pain intensity and opioid use after surgery.^28^

The Opioid Risk Tool, which can be easily sourced on the Internet,^29^ is a questionnaire that is usually used to assess an adult’s propensity to develop an opioid use disorder in the setting of chronic pain. It uses a point system to stratify risk based on factors such as a history of personal or family drug abuse, age, psychiatric history, and a history of childhood sexual abuse. It may be awkward to screen each patient coming for surgery for such issues, but in the case of high suspicion (such as high requirements for opioids) postoperatively, use of the tool may be appropriate.

2.1.1. If a patient is at risk for persistent opioid use, a tailored opioid-sparing perioperative pain management plan should be developed by the perioperative team (including surgeons, anesthesiologists, and/or the pain team) preoperatively.

The perioperative period provides a critical window to address opioid use, particularly in patients with a history of chronic pain and presurgical opioid use. No other factor is as consistently related to the development of future pain problems as is concurrent pain.^30^ Therefore, patients with a pain problem taking opioids prior to surgery (approximately 12.5% of the surgical population) could benefit from specialized pain services.^31^ The current consensus statement is not suited to patients already consuming opioids prior to surgery. As noted above, extra care should be taken for procedures known to be associated with a higher risk of persistent opioid use. Clarke et al. found that accounting for all demographic and comorbid factors, patients who underwent thoracic surgery procedures had a 2.5-fold higher risk of requiring opioids long term compared to patients having any other surgical procedure.^23^ Sun et al., using U.S. administrative health claims databases, found that patients who had surgery had a higher persistent use of opioids 1 year after surgery compared to nonsurgical patients.^32^ At 1 year, 1.4% individuals who had had a total knee arthroplasty were taking opioids, compared to 0.136% of nonsurgical patients.^32^

Specialized care for individuals that comprehensively addresses the potential problems of patients at high risk of persistent postoperative opioid use should occur throughout the perioperative time period: (1) preoperatively, (2) postoperatively in hospital, and (3) postoperatively in an outpatient setting and should be modeled at institutions that offer major surgical interventions.^24^ Following presurgical assessment, patients about to undergo higher risk surgeries that have a higher incidence of chronic pain and/or persistent opioid use should be assessed by a specialized pain service/practitioner.^33, 34^

3. Discharge prescriptions for pain management after elective surgery
  3.1. Nonopioid therapy should be the first-line of treatment for pain. Therapies can be pharmacological (e.g., nonsteroidal anti-inflammatory drugs [NSAIDS], acetaminophen, or regional anesthetic techniques) or nonpharmacological (e.g., physical therapy, ice/heat, elevation, breathing exercises, meditation, etc.).

The World Health Organization’s pain management ladder is the model followed generally around the world in the postoperative setting. This model suggests the use of nonopioids such as NSAIDS and acetaminophen prior to introducing weak opioids and then stronger opioids.^33^

There is significant literature to support multimodal analgesia (given the synergistic effects of different classes of medications) rather than sole opioid use with recognition that reduced opioid doses are needed in patients receiving nonopioid analgesia (a concept known as *opioid sparing* that has been recognized for decades).^35–44^ In 2018, Memtsoudis et al., after a retrospective analysis of 1,540,462 arthroplasties, demonstrated an inverse relationship between the number of nonopioid modalities employed and total dose of opioids used postsurgically.^45^

There are many reviews and large studies on multimodal analgesia using widely available and affordable options such as acetaminophen and NSAIDs.^46^ Some studies have also demonstrated synergism when these are used together.^47,48^ Furthermore, adjunctive options such as gabapentinoids have been demonstrated to be opioid sparing, in particular when a neuropathic pain component is involved.^49–51^ Low-dose parenteral and oral ketamine is also becoming part of the armamentarium of nonopioid analgesics.^52,53^

Interventional pain medicine has been recently expanding its scope to include myofascial plane blocks that are proving to be both versatile and efficacious. The erector spinae and transversus abdominus plane blocks, which directly block intercostal nerves that exit the spinal cord, have recently become popular options for several procedures, providing advantages of technical simplicity and reduced opioid intake.^54–57^ The use of peripheral nerve blocks is also an established form of pain control that has proven efficacy in the management of postoperative pain. The application of local anesthetics into the surgical site to reduce pain and opioid requirements in the postoperative setting has demonstrated efficacy and been supported for decades by surgeons.^58,59^ The use of such interventional techniques is best determined by the anesthesia staff, who can assess the appropriateness of an intervention in each case and whether they are equipped with the needed training and tools to render the service.

Although extensive studies on nonpharmacologic therapies such as breathing techniques, cold therapy (ice packs), and acupuncture are lacking, these modalities are thought to inhibit pain signals by activating neurons in the spinal cord that directly block the conduction of such signals. In the case of cold therapy, slowing of signal conduction is also proposed,^60^ whereas for acupuncture, modulation of the endogenous opioid system is thought to be one of several underlying mechanisms.

Of all of the techniques, acupuncture’s supportive body of evidence is the largest and includes mostly positive findings as found through meta-analyses and systematic reviews. Low numbers of studies of heterogenous design and quality limit the reliability of data, however, and more studies are needed to reach a consensus.^61–64^ Evidence for cold therapy exists so far only in randomized controlled trials for a variety of surgical procedures, including hernia repair, A-V fistula creation, and dental surgery.^60,65–73^ Other methods also need further study even though they are endorsed by various institutions.^74,75^

Lack of movement postoperatively can lead to muscle tightness and pain. Physiotherapists work with patients to alleviate kinesiophobia and restore function using multiple tools. Stretching, acupuncture, scar tissue massage, myofascial release, exercise prescription, education on using mobility aids, and/or a prescription regarding using certain equipment in patients daily lives provide the basis of good postoperative physiotherapy care.

3.2. Patients should be discharged with a prescription for the following adjunct pain medications unless contraindicated:
• Acetaminophen 1 g PO TID to QID for 7 days then PRN.
• NSAIDS (e.g., naproxen 500 mg BID or ibuprofen 400 mg QID) PO for 3 days then PRN.
3.2.1. Patients should be counseled on how to take scheduled medications and advised to stop taking these medications after 7 days if they are expected to have a rapid recovery or after 14 days if they are expected to have a moderate or long-term recovery.

Acetaminophen and NSAIDS are fundamental starting points for pain management in the hierarchy of analgesic drugs. The World Health Organization’s guidelines for acute pain management state that these two drug categories should be first-line pharmacologic agents, with opioids being additive, if there is need for further analgesia.^76^ They have a lower profile of adverse effects in comparison to opioids, and their cost and availability are other advantages. Memtsoudis et al. have further reinforced their value, showing that of all the nonopioid pharmacologic analgesic modalities, NSAIDS are the most effective at reducing opioid consumption for orthopedic joint surgery.^45^

Sole acetaminophen therapy has been shown to relieve moderate pain for several hours while decreasing the overall dose of opioids.^77^ When used together with NSAIDS, however, both drugs are more effective than when used on their own.^78,79^

There has been debate in the past about possible renal impairment and postoperative bleeding with the use of NSAIDS, but systematic reviews have shown these drugs to be nonthreatening to normally functioning kidneys and benign with respect to hemostasis.^80,81^ Caution is suggested for patients over 75 years old, however, because gastrointestinal and surgical site bleeding has been shown to occur in this age group. Caution is also advised for patients with colonic anastomoses because studies have suggested increased anastomotic leakage with NSAID use. In such cases, use should be at the surgeon’s discretion.^80,82^ Care must also be taken not to exceed the recommended dosages of each drug and to always rule out contraindications before starting. Contraindications to NSAID use include peptic ulcer disease, gastroesophageal reflux disease, inflammatory bowel disease, bleeding abnormality, congestive heart failure, uncontrolled hypertension, pregnancy in the third trimester, and a history of NSAID-associated asthmatic attack. NSAIDs should also be avoided in patients on drugs that may interact with them, such as anticoalgulants and cyclosporine. Celecoxib can be substituted for an NSAID if there is a history of peptic ulcer disease.

There continues to be debate within the orthopedic literature regarding the impact of NSAIDs on bone healing, with concern about the potential for increased rates of nonunion. Though the literature is not conclusive, a recently published meta-analysis reported increased odds of nonunion (odds ratio = 2.07, 95% CI, 1.19 to 3.61) in orthopedic patients taking high doses or long courses of NSAIDs following fracture osteotomy and fusion surgeries.^82^ In this study, low doses of NSAIDs (less than 125 mg/day diclofenac, 150 mg/day indomethacin, or 120 mg/day ketorolac) taken for less than 2 weeks were not shown to increase the odds of nonunion.^83^ Several other studies also suggest that short courses of NSAIDs do not affect nonunion rates.^84,85^ However, no sufficiently powered randomized controlled trial has yet been performed to definitively prove whether or not NSAIDs affect bone healing. It is therefore suggested that preference be given to short courses of low-dose NSAIDs following orthopedic procedures where there is a risk of nonunion (e.g., fracture, osteotomy, and fusion procedures) and that consideration be given to avoiding NSAIDs in patients at high risk of nonunion.

Interestingly, a recent study has also implicated opioid use in the development of fracture nonunion among trauma patients, with the odds of nonunion being higher for most of the opioid medications when compared with acute NSAID use.^86^ This may provide additional support for the use of short courses of low-dose NSAIDs even in patients at risk of nonunion, if this permits reduced opioid use.

Scheduled dosing is recommended in order to maintain drug levels and thereby the analgesic and opioid-sparing effects.^87^ The scheduled acetaminophen course duration should mirror the opioid course duration. NSAID scheduled dosing is advised for 3 days by our panel, with the intent to avoid side effects such as gastritis and gastric ulceration, which are more likely with prolonged use of nonselective cyclooxygenase inhibitors. Prescribers, nurses, and pharmacists should counsel patients on how to take scheduled medications (e.g., while awake) and educate patients that concomitant pain medications can be taken together.

3.3. Patients should receive a prescription for opioid-containing tablets based on their consumption in hospital during the previous 24 h.
  3.3.1. Patients should receive the same opioid analgesic that they received in hospital to ensure tolerability.
  3.3.2. Patients who did not receive opioids in the last 24 h of their hospital stay should not be given a prescription for opioids.
  3.3.3. Day surgery patients should be prescribed medications for patients with an expected rapid recovery as illustrated in Table 1.

**Table 1.** Examples of surgical procedures and their expected recovery times

	Expected rapid recovery	Expected moderate recovery	Expected long-term recovery
Definition	Patient resumes most normal activities within 2 weeks from surgery	Patient resumes most normal activities within 4 weeks from surgery	Patient resumes most normal activities after 4+ weeks from surgery
Number of opioid-containing tablets	0–3 days; maximum 12 tablets	7 days; maximum 30 tablets	14 days; maximum 60 tablets; split prescription (see Appendix)
Breast procedures	● Breast biopsy ● Lumpectomy (with or without sentinel lymph node biopsy) ● Sentinel lymph node biopsy ● Simple mastectomy (with or without sentinel lymph node biopsy)	● Mastectomy with reconstruction ● Modified radical mastectomy ● Axillary lymph node dissection	
Cardiac procedures	● Cardiac catheterization	● CABG	
General surgery procedures	● Cholecystectomy ● Appendectomy ● Inguinal/femoral hernia repair (open/laparoscopic) ● Umbilical hernia repair (open) ● Ileostomy/colostomy Cceation (lap) ● Colon or rectal resection (lap)	● Ileostomy/colostomy creation (open) ● Incisional hernia repair (lap or open) ● Small bowel resection or enterolysis (open) ● Low anterior resection (lap or open) ● Colon or rectal resection (open) ● APR	● Component separation and incisional hernia repair
Gynecological procedures	● Uncomplicated cesarean section ● Uncomplicated labor and delivery	● Vaginal hysterectomy ● Abdominal/open hysterectomy ● Laparoscopic and robotic hysterectomy	
Neurosurgery and spine procedures	● Microdiscectomy (one level)	● Discectomy (open/multilevel) ● Laminectomy ● Craniotomy	● Lumbar fusion, major spine procedure
Orthopedic procedures	● Arthroscopic partial meniscectomy ● Carpal tunnel release ● Acute fracture (closed reduction, no surgery) ● Minor fracture ORIF (e.g., wrist, ankle, foot, patella, olecranon) ● Arthroscopic shoulder decompression	● Arthroscopic ACL/PCL reconstruction ● Arthroscopic or mini open rotator cuff repair ● Thumb reconstruction ● MTP fusion ● Major fracture ORIF (e.g., humerus, tibia, tibial plateau, pilon, femur) ● Hip fracture (ORIF or arthroplasty) ● Total hip arthroplasty ● Total shoulder or elbow arthroplasty ● Total ankle arthroplasty ● Amputation	● Total knee arthroplasty ● Osteotomies ● Revision surgeries for fracture nonunion ● Repair/reconstruction of multiligament knee injuries
Otolaryngology procedures	● Thyroidectomy, tonsillectomy ● Cochlear Implant		● Partial or complete neck dissection
Thoracic procedures	● VATS (video-assisted surgery: lobectomy/wedge resection, thymectomy)	● Thoracotomy	● Esophagectomy
Urological procedures	● Robotic retro pubic prostatectomy ● Vasectomy ● Transurethral resection of bladder tumor ● Ureteral stent placement ● Ureteroscopic stone extraction	● Robotic-assisted laparoscopic radical prostatectomy ● Robotic assisted laparoscopic partial nephrectomy ● Percutaneous nephrectomy ● Inflatable penile prothesis/malleable penile prothesis placement	● Open partial nephrectomy ● Open cystoprostatectomy with ileal conduit
Vascular procedures	● Endovascular thoracic/aortic aneurysm repair (EVAR/TEVAR) ● Upper extremity dialysis access creation ● Carotid endarterectomy	● Infrainguinal bypass ● Hybrid infrainguinal revascularization ● Thoracic outlet decompression ● Advanced endovascular aortic aneurysm repair (fEVAR, P-branch)	● Open aortic aneurysm repair ● Open thoraco-abdominal aneurysm repair ● Aorto/thoraco–femoral bypass

ORIF = open reduction and internal fixation; ACL = anterior cruciate ligament; PCL = posterior cruciate ligament; MTP = First metatarsal-phalangeal; APR = abdominal perineal resection; VATS = video-assisted thorascopic surgery; CABG = coronary artery bypass graft; EVAR = endovascular aortic repair; TEVAR = thoracic endovascular aortic repair; fEVAR = femoral endovascular aortic repair.

**Table 2.** Oral opioid analgesic conversion table

	Equivalence to oral morphine 30 mg	To convert to oral morphine equivalent, multiply by	To convert from oral morphine, multiply by
Morphine	30 mg	1	1
Codeine	200 mg	0.15	6.67
Oxycodone	20 mg	1.5	0.667
Hydromorphone	6 mg	5	0.2
Methadone and tramadol	Morphine dose equivalence not reliably established		

Source: Canadian Guideline for Safe and Effective Use of Opioids for Chronic Non-Cancer Pain (http://nationalpaincentre.mcmaster.ca/opioid/cgop_b_app_b08.html).

Theisen et al. recently highlighted research on opioid overprescribing, recording a range of 37% to 71% of opioid pills prescribed being unused in the postoperative discharge period for various surgery types.^88^ Some studies have even found that a proportion of patients use no opioids postdischarge, with studies citing no postdischarge opioid use in 82% of partial mastectomies, 41% of partial mastectomies with sentinel node biopsy, 35% of laparoscopic cholecystectomies, 45% of laparoscopic inguinal hernia repairs, and 22% of open inguinal hernia repairs.^89–93^

Overprescribing by surgeons has been called into question with respect to fueling the opioid crisis. Studies have shown that even a single prescription can be linked to chronic use regardless of surgery type and that up to 80% of heroin addicts were first introduced to opioids through prescriptions,^88,94^ although they were not necessarily prescribed to them.

In view of the various dangers posed, our panel recommends a personalized approach for prescribing opioids in the postdischarge period.^95,96^ A practical means of deciding “how much” is to check the patient’s in-hospital usage within the last 24 h and use this as a guide to determine the daily prescribing dose. Osmundson et al. compared this approach to a standardized opioid prescription of 30 oxycodone 5 mg pills given to women post cesarean section.^95^ Of the 127 women included, those given individualized perscriptions were prescribed less opioids and used half the amount of the opioids prescribed compared to those who were given the standard prescription. Those not needing opioids in hospital were not prescribed any opioids postdischarge.^96^

Using the final 24 h for prescribing estimation was suggested by Hill et al. after analyzing 333 cases of a wide range of operations, including minor procedures such as inguinal hernia repair and major ones such as colon surgery.^97^ Hill et al. went on to recommend 15 opioid-containing pills for patients using one to three pills, 30 for those using four or more pills, and none for those not requiring any in the 24 h prior to discharge. Their mathematical model is designed to cover analgesic needs while keeping opioid prescribing to a minimum.^96^ Our panel’s final recommendation takes into consideration further models of prescription estimation as well as duration of therapy.

Despite published tables suggesting conversion factors to be used in calculating equipotent doses for different opioids, we strongly suggest prescribing the same opioid that the patient used while in hospital. Conversion tables are variable and the conversions can be unreliable. Opioids also have different receptor affinities, and this is complicated further by interindividual variability in opioid metabolism and sensitivity and concurrent patient comorbidity. At present, pain specialists are guided by a two-step calculation derived by Fine and Partenoy when switching opioids.^98^ This is still meant to be used merely as a guide and not representative of absolute conversion values (see Table 2 as a commonly used opioid conversion calculator). A pharmacist can be consulted for opioid equivalents conversion if the patient needs to switch opioid therapy due to intolerance or side effects. For matters of safety and simplicity, one should adhere to a single opioid for in- and out-of-hospital use.^99,100^

3.4. Prescriptions for opioid-containing tablets should be written during the discharge process. Prescriptions should not be written in advance (i.e., prior to surgery) for surgical inpatients.

The standardization of processes can be an attractive concept adopted in an effort to seek simplification and speed on a busy surgical unit. In some cases, discharge prescriptions may be written as part of a preparatory package, even before the procedure takes place.

Though this may appear to improve efficiency, its practice in the current context of an opioid crisis is discouraged. One should keep in mind that opioids bear high street value and are often subject to diversion.^101^ Supplying a patient with more opioids than is needed is a possibility when prewriting prescriptions and can potentially contribute to diversion.

An analysis of all opioid related deaths at St. Michaels Hospital, Toronto, in 2016 showed that of all deaths among those with no active opioid prescription, 18% tested positive for hydromorphone, 17% for oxycodone, and 17% for morphine.^102^ One study even cited an estimate of 71% of diverted drugs being acquired through family or friends.^103^

Writing an opioid prescription without knowing the exact dose required by a patient may represent a “license” for more drugs than needed and open a pathway to drug access and diversion. One news report stated that nearly 9 million doses of prescription drugs were reported “missing” between 2012 and 2017 in Canada, with the majority of the drugs being opioids. This information alone serves as a good indication of the level of caution that needs to be taken with opioid prescription and even unused prescription pads.^101^

Waiting for the day of discharge and making a calculated decision on exactly how many opioids to prescribe is the best way to meet the analgesic needs of the patient while protecting the public from unnecessary exposure to the drugs.

3.5. Patients who are given a prescription for opioid-containing tablets should be instructed to fill the prescription only if their pain is not well managed with other therapies or if they are having difficulty completing activities of daily living secondary to pain.

In the past, no pain in the postoperative recovery period was regarded by some institutions as the theoretical “gold standard” for patients. With the increase of regional anesthesia techniques, “no pain” may be achievable in a subset of patients; however, many do not have access to these techniques, and the chase for “no pain” has likely provided impetus to the overprescribing of opioids.^104^

The goal of pain management in the postoperative period is to keep the patient comfortable enough (i.e., pain intensity below 4 on a 0–10 scale) to maintain functionality.^105^ Though no pain would be ideal, it is unreasonable to expect that someone just having received a major incision will have absolutely no discomfort even after receiving painkillers.

Recognizing the need for redefinition of the goals of pain management in order to stem the tide of opioid overprescription and addiction, the U.S. Joint Commission developed 19 elements of performance that accredited national hospitals were expected to meet by January 2018. Proper control of pain was emphasized to be reflected in a patient’s ability to achieve functionality goals. Goals in this case are dynamic concepts and change from day to day depending on expected ability. Taking full breaths comfortably on the first day following major abdominal surgery, for example, would be acceptable, as opposed to walking to the bathroom comfortably a few days later.^106^

Australia and New Zealand have also been incorporating a “functional activity scale” in pain assessments, and Great Britain uses the “dreaming” concept or the general ability to drink, eat, and mobilize in the postoperative period.^107,108^

Regardless of the school of thought, the principle remains the same: Less emphasis on traditional pain scores and more focus on function is the rational approach to assessment. Expectations of pain management should be set out from the very inception at the preoperative consult, and patients should be told that as long as they can function, within reason, with nonopioids postdischarge, they should not fill a prescription for opioids that might have been given.

3.6. Opioid prescribing should be based on expected functional recovery (provided the patient has not exceeded the dose below in the past 24 h if applicable) See Table 1 for expected recovery times for common surgical procedures.
  3.6.1. Patients with an expected rapid recovery (resume regular activities within 2 weeks from discharge) should be prescribed enough opioid-containing tablets for 0–3 days following discharge (maximum 12 opioid-containing tablets).
  3.6.2. Patients with an expected moderate recovery (resume regular activities within 4 weeks from discharge) should be prescribed opioid-containing tablets for a maximum of 7 days following discharge (maximum 30 opioid-containing tablets).
  3.6.3. Patients with an expected long-term recovery (resume regular activities longer than 4 weeks from discharge) should be prescribed opioid-containing tablets for a maximum of 14 days following discharge (maximum 60 opioid-containing tablets).
  3.7. A part-fill or second prescription should be given to patients with an expected moderate or long term recovery (recommendations 3.6.2 and 3.6.3) to reduce the number of opioid-containing tablets distributed at one time (see Appendix for sample prescriptions).

There are two main variables in opioid prescribing: One is the total opioid dose used for the entire course postdischarge and the second is the number of days that the opioids are used. Both Shah and Bratt have published evidence that links duration of opioid use with later chronic use^109^.

Shah et al. noted an incidence of long-term use (use for more than 1 year) in 13.5% of persons whose first episode of use was for ≥8 days (for various types of acute pain), and this increased to 29.9% when the first episode of use was for ≥31 days.^110^ Brat et al. found that each refill and additional week of opioid use was associated with an increase in the rate of misuse by 44.0%.^1^ Although Shah et al. related long-term use to initial high doses of opioid (700 mg cumulative oral morphine equivalents), Sekhri et al. noted that the number of refill requests was not linked to this factor.^111^ Thus, the length of the first opioid exposure seems to be the underlying factor linked to chronic use.

To date, there is no global consensus on the length of the duration of therapy for each surgical procedure. Scully et al., however, tracked general trends in opioid needs for more than 200,000 patients following several operation types in an effort to propose a reference point for initiating therapy.^109^ They theorized that the optimum duration of therapy was between the observed median prescription length and the earliest discontinuation of use of refill prescriptions. Using this model, 4 to 9 days for general surgery procedures, 4 to 13 days for women’s health procedures, and 6 to 15 days for musculoskeletal procedures were suggested as the average initial lengths of opioid prescription.^112^

With respect to the number of pills to be prescribed, the use of the last 24-h period in hospital to estimate the number of prescribed pills as espoused by Osmundon et al.^95^ and Hill et al.^96^ was discussed previously. Theils et al. also performed an extensive multicenter analysis on opioid requirements following 25 common surgical procedures, leading to the Mayo Clinic’s detailed outline of prescribing low-, standard, and high-dose regimens, including separate guidelines for orthopedic surgery.^93^

After comparative review of available data and discussion that incorporated longstanding experience in the field, our panel recommends the above as a guide to initial opioid prescribing in the postdischarge period. We believe that this guide will serve to balance the analgesic needs of most patients with the societal need to keep opioid exposure at a minimum. The consensus statement should be used in accordance with the categorization of various procedures in Table 1.

3.8. The prescription for opioid-containing tablets should have an expiry date of 30 days from the date of discharge (see Appendix for a sample prescription).

According to Canadian law, as long as a prescription bears no expiry date, it may be filled within 1 year of the date it was written.^113–115^ This leaves a gaping loophole for the legal acquisition of opioids that were prescribed but for which the prescription was not filled for the purpose of postoperative pain management.

This represents a dangerous situation, and until laws are readjusted, our panel recommends clear indication of expiry of an opioid prescription 30 days after it is issued. A 1-month period should be adequate for a patient to assess the need for the supplementary medication. If severe pain reemerges after this period, a consult with one’s surgeon for reassessment would be appropriate, and any needed prescriptions could be reissued at this point. Implementation of this practice is intended to effect reduction in the amount of circulating opioids available for diversion.

3.9. If opioid-containing tablets are prescribed, they should be short-acting opioids at the lowest effective dose, with the lowest potency, for the shortest duration. Patients should be prescribed one of the following opioid-containing tablets:
  • Morphine 5 mg, PO q4h PRN for 3 days then q8h PRN.
  • Hydromorphone 1 mg, PO q4h PRN for 3 days then q8h PRN.
  • Tramadol 50 mg, PO q4h PRN for 3 days then q8h PRN.
  • Oxycodone 5 mg, PO q4h PRN for 3 days then q8h PRN.

*Oxycodone and tramadol are not covered by the Ontario Drug Benefit and will be paid out of pocket by patients.

In 2016, the Centers for Disease Control issued guidelines on opioid use for chronic pain and advised: “When opioids are used for acute pain, clinicians should prescribe the lowest effective dose of immediate-release opioids and should prescribe no greater quantity than needed for the expected duration of pain severe enough to require opioids. Three days or less will often be sufficient; more than 7 days will rarely be needed.”^116^

The pharmacokinetics of different drug formulations justify the use of immediate-release rather than continuous-release formulations. Immediate-release opioids usually have a peak effect at 45 to 60 min as opposed to continuous-release formulations, which peak at around 3 h. The use of immediate-release opioids therefore affords the user quicker assessment of the effect with the ability to titrate the dose within a reasonable amount of time. Waiting for longer-acting opioids to have their full effect may encourage patients who are in pain to stack doses to their detriment.

In 2015, Miller et al. published a study of patients prescribed opioids in the United States using the Veterans database. After propensity score matching, they found that those prescribed long-acting opioids on initiation of therapy were twice as likely to experience accidental overdose than those prescribed short-acting opioids. The overdose events were noted to be most frequent in the first 2 weeks of therapy, no doubt likely associated with troubleshooting dose titration.^117^

The above dosages represent estimated equi-analgesic amounts of opioids that are currently available in Canada. As discussed previously, these doses do not represent perfect values but are rough figures using multiplication coefficients derived from a debatable and relatively small database. No current evidence exists at present to suggest prescription of one opioid in preference to another, although we do know that interpatient variability due to genetic differences in receptors and opioid affinity is a reality. Ultrarapid metabolism of codeine to morphine (in people who carry the gene that facilitates this process) can lead to inadvertent overdose.^118^ Codeine was therefore not included in the list of suggested opioids.

Our panel suggests the above as a guide only for healthy opioid-naïve adults. As mentioned before, opioid prescribing should be individualized and take into consideration factors such as patient comorbidity, age, and weight when making a final decision.

4. Follow-up and long-term opioid use
  4.1 Surgeons should ask their patients at follow-up about their postoperative pain and opioid use. Patients should be instructed to return unused opioids to their local pharmacy at the time of their follow-up if they are not being used.

It is incumbent upon all opioid-prescribing physicians to monitor their patients’ opioid use. It is expected that as healing continues, the dose of opioids needed would become progressively less. A lack of decreasing need for opioids or an increasing need for them may indicate early signs of a persistent pain problem, addiction, tolerance, or another medical issue that should be addressed. Shah et al. documented that the probability of long-term opioid use increases significantly at the second prescription for opioids and in patients on their 5th to 31st day of opioid therapy.^110^ The surgeon should therefore be on the alert when a second or third opioid prescription is requested.

The role of the pharmacist at the time of filling the postdischarge prescription is important. Pharmacists should discuss opioid equivalents (i.e., safe consumption guidelines) and interactions with other medications (i.e., benzodiazepines and anticonvulsants).

At present, opioid risk assessment tools such as the Pain Medication Questionnaire and the Screener and Opioid Assessment for Patients with Pain are used mainly in the setting of chronic pain but may still be used to give an idea of a patient’s propensity for misuse.^119^ The Current Opioid Misuse Measure may also be helpful, as may be urine screens to check that the patient is actually using the medication and not diverting. Studies are still ongoing to refine tools to assist with risk assessment, especially in the acute care setting.^120^

Unused opioids should never be kept by patients. Unused opioids should be returned to the dispensing pharmacy. In addition to the possibility of entering public circulation, contamination of soil and water due to poor disposal techniques is becoming an environmental issue. Pediatric exposure is also a possibility with unfortunate consequences. From 2000 through 2015, 188,468 pediatric opioid exposures were reported to U.S. poison control centers, and thousands of opioid-related deaths among minors have been recorded.^121,122^ Alarming statistics such as these are congruent with Theisen et al.’s report of anywhere between 53% and 91% of patients being found to have kept leftover opioids postrecovery.^88^ In 2016, Kim et al. also reported a rate of only 5.3% of patients being instructed on what to do with unused opioids after orthopedic surgery,^123^ and Bartels et al. found that 77% of post–C-section patients and 73% of post–thoracic surgery patients in their study stored unused opioids in an unlocked location.^91^

4.2. If patients require a refill prior to their scheduled follow-up visit, a maximum of 14 days (maximum 60 opioid-containing tablets) should be prescribed. Dated part fills for 7-day intervals should be given.

In a subset of patients, acute pain evolves into a chronic pain condition. It is estimated that the 1-year incidence of moderate to severe chronic postsurgical pain is between 5% and 10%.^124^ Chronic pain in this context is defined as pain lasting for 12 or more weeks following surgery and is mediated by neural rearrangement and dysfunction. The incidence of opioid misuse disorders among chronic pain sufferers is debatable, with a wide range from 0.5% to 81%.^125^ Longstanding pain is still described as a potential^115^ predictor of future opioid misuse.^126–128^

In patients with chronic postsurgical pain, the incidence of opioid misuse is up to 10% 1 year following surgery.^33^ Factors predicting it have been well defined and include preoperative pain and opioid use, the extent of trauma surrounding the perioperative course, and a background of psychiatric mood or anxiety disorders.

4.3. If pain persists beyond 3 months, patients should be referred to a transitional/chronic pain clinic.
  4.4. If there is a suspicion that a patient is misusing opioids, the patient should be referred to a transitional/chronic pain clinic.

Chronic postsurgical pain has an incidence of up to 10% 1 year following surgery.^33^ Factors predicting it have been well defined through research and include preoperative pain and opioid use, the extent of trauma surrounding the perioperative course, and a background of psychiatric mood or anxiety disorders.

The role of the transitional pain clinic is to essentially change the trajectory of acute pain in order to avert the development of chronic pain syndromes and their associated negative entourage, including opioid use disorders. Patients identified as “high risk” can be referred preoperatively and then followed postoperatively. Patients who did not appear to be at high risk but seem to have persisting pain beyond the expected postoperative period should also be referred.

A biopsychosocial approach is the core strategy of this clinic. The Toronto General Hospital’s model incorporates an anesthesiologist with specialized training in pain medicine, advanced practice nurses, psychologists, a pharmacist, a physiotherapist, and a yoga practitioner.

In addition to improving patient coping and functioning, the primary focus of this clinic is responsible surveillance of opioid use and opioid weaning postoperatively. Preliminary data show rates of up to 46% and 26% complete weaning in opioid-naïve and opioid-experienced patients, respectively, 6 months postsurgery through The Toronto General Hospital’s Transitional Pain Clinic.^24,33^ Surgeons should make full use of such services not only as part of good clinical practice but also as part of exercising social responsibility in an era of prescription drug abuse. Institutions that provide major surgical interventions should mandate programs that provide support to patients with complex postsurgical.

## Limitations

1. Limited evidence is currently available on the subject of perioperative opioid harm reduction strategies and their effectiveness. The impact of implementation on workers’ time and institution funds is also unknown. Undertaking analysis of the effects postimplementation of this consensus statement will be necessary to validate these statements and their impact on perioperative care.
2. This document does not outline exactly which professionals should hold which responsibilities. Education, for example, may be the responsibility of the primary surgery team in some institutions, whereas it may be that of the pharmacists, preoperative education teams, and/or nurse practitioners in others. Each institution will need to designate roles according to their local custom and resources.
3. The extent to which institutions apply these recommendations will vary according to resources.

## Future Research

1. Postimplementation metrics will focus on how well this information was disseminated and whether an overall reduction in opioids prescribed at discharge was implemented with a concomitant measure of patient satisfaction and global functioning.
2. As other groups create similar statements and academic material accrues on strategies for postoperative opioid prescribing and pain management, these documents should be graded to lead to the creation of a postoperative opioid prescribing guideline that would be acceptable to a wide audience.
3. This consensus statement is not intended to apply to certain populations; for example, pregnant women, children, patients with chronic pain, or patients already on opioids preoperatively. It is therefore limited in its application. Ideally, future versions of this document will incorporate guidance for the excluded populations, particularly patients with chronic pain on opioid medications preoperatively and the pediatric population.
4. The effect of this consensus statement on postoperative pain and long-term functioning is not known. Prospective cohort studies are required to assess these long-term outcomes.

## Appendix. Examples of postoperative for pain medication at discharge from elective surgery

Name: JOHN DOE

Date of issue: January 1, 2020

**Rapid Recovery Example**:

1. Acetaminophen 1 g PO TID × 7 then PRN

2. Ibuprofen 400 mg PO QID × 3 days then PRN

3. Morphine 5 mg tabs

Take 1–2 tabs q 4 h PRN × maximum of 3 days for severe pain

Maximum 4 tablets/day

Dispense quantity: 12 tabs

Prescription expires 30 days after date of issue

**Moderate Recovery Example**:

1. Acetaminophen 1 g PO TID × 14 days then PRN

2. Ibuprofen 400 mg PO QID × 6 days then PRN

3. Hydromorphone 1 mg tabs

Take 1–2 tabs q 4 h PRN × maximum of 14 days for severe pain

Maximum 4 tablets/day

Dispense interval: Dispense 15 tablets now and 15 in 3 days

Prescription expires 30 days after date of issue

**Long-Term Recovery Part Fill Example**:

1. Acetaminophen 1 g PO TID × 14 days then PRN

2. Ibuprofen 400 mg PO QID × 6 days then PRN

3. Hydromorphone 1 mg tabs

Take 1–2 tabs q 4 h PRN × maximum of 30 days for severe pain

Maximum 4 tablets/day

Dispense interval: Dispense 30 tablets now and 30 in 7 days

Prescription expires 30 days after date of issue
